# Greater aortic inflammation and calcification in abdominal aortic aneurysmal disease than atherosclerosis: a prospective matched cohort study

**DOI:** 10.1136/openhrt-2019-001141

**Published:** 2020-03-11

**Authors:** Nikhil V Joshi, Maysoon Elkhawad, Rachael O Forsythe, Olivia M B McBride, Nikil K Rajani, Jason M Tarkin, Mohammed M Chowdhury, Emma Donoghue, Jennifer M J Robson, Jonathan R Boyle, Tim D Fryer, Yuan Huang, Zhongzhao Teng, Marc R Dweck, Ahmed A Tawakol, Jonathan H Gillard, Patrick A Coughlin, Ian B Wilkinson, David E Newby, James H F Rudd

**Affiliations:** 1 Centre for Cardiovascular Science, University of Edinburgh, Edinburgh, UK; 2 Division of Cardiovascular Medicine, University of Cambridge, Cambridge, UK; 3 Department of Vascular Surgery, University of Cambridge, Cambridge, United Kingdom; 4 Wolfson Brain Imaging Centre, University of Cambridge, Cambridge, United Kingdom; 5 Harvard University, Cambridge, Massachusetts, USA; 6 Department of Medicine, University of Cambridge, Cambridge, United Kingdom

**Keywords:** AAA, PET-CT, aneurysms, atherosclerosis

## Abstract

**Objective:**

Using combined positron emission tomography and CT (PET-CT), we measured aortic inflammation and calcification in patients with abdominal aortic aneurysms (AAA), and compared them with matched controls with atherosclerosis.

**Methods:**

We prospectively recruited 63 patients (mean age 76.1±6.8 years) with asymptomatic aneurysm disease (mean size 4.33±0.73 cm) and 19 age-and-sex-matched patients with confirmed atherosclerosis but no aneurysm. Inflammation and calcification were assessed using combined 18F-FDG PET-CT and quantified using tissue-to-background ratios (TBRs) and Agatston scores.

**Results:**

In patients with AAA, 18F-FDG uptake was higher within the aneurysm than in other regions of the aorta (mean TBR_max_2.23±0.46 vs 2.12±0.46, p=0.02). Compared with atherosclerotic control subjects, both aneurysmal and non-aneurysmal aortae showed higher 18F-FDG accumulation (total aorta mean TBR_max_2.16±0.51 vs 1.70±0.22, p=0.001; AAA mean TBR_max_2.23±0.45 vs 1.68±0.21, p<0.0001). Aneurysms containing intraluminal thrombus demonstrated lower 18F-FDG uptake within their walls than those without (mean TBR_max_2.14±0.43 vs 2.43±0.45, p=0.018), with thrombus itself showing low tracer uptake (mean TBR_max_ thrombus 1.30±0.48 vs aneurysm wall 2.23±0.46, p<0.0001). Calcification in the aneurysmal segment was higher than both non-aneurysmal segments in patients with aneurysm (Agatston 4918 (2901–8008) vs 1017 (139–2226), p<0.0001) and equivalent regions in control patients (442 (304-920) vs 166 (80-374) Agatston units per cm, p=0.0042).

**Conclusions:**

The entire aorta is more inflamed in patients with aneurysm than in those with atherosclerosis, perhaps suggesting a generalised inflammatory aortopathy in patients with aneurysm. Calcification was prominent within the aneurysmal sac, with the remainder of the aorta being relatively spared. The presence of intraluminal thrombus, itself metabolically relatively inert, was associated with lower levels of inflammation in the adjacent aneurysmal wall.

**Key questions**

**What is already known about this subject?**

- Although the biology of aortic aneurysm and atherosclerosis remains incompletely understood, both diseases share common risk factors and pathological features. Inflammation and calcification of the aneurysm wall play a key role in initiation, progression and destabilisation in abdominal aortic aneurysm (AAA). Retrospective studies have been undertaken to assess inflammation and calcification in aneurysm subjects; however, prospective studies using state-of-the-art imaging protocols, and comparison with matched atherosclerotic controls is lacking.

**What does this study add?**

- This is the first study to measure both inflammation and calcification in patients with aneurysm and to compare it with age-and sex matched controls with atherosclerosis. The study adds several important mechanistic insights into the pathobiology of AAA. We observed that both aortic inflammation and calcification are greater in patients with aortic aneurysm than those with atherosclerosis alone, that aortic inflammation typically extends beyond the aneurysmal sac to involve the entirety of the aorta and that greater thrombus burden is associated with less inflammation.

**How might this impact on clinical practice?**

- We have demonstrated heightened inflammation extending beyond the aneurysmal segment in those with small to medium sized aneurysms. Prospective studies are now needed to evaluate the (1) the prognostic value of measuring aortic inflammation and calcification to improve clinical decision-making in patients with asymptomatic AAA and (2) whether anti-inflammatory agents may reduce aneurysm formation and expansion.

## Introduction

Abdominal aortic aneurysm (AAA) is a matrix-degenerative vascular disorder resulting in aortic dilatation (diameter >3 cm), with a prevalence of around 5% in adults aged 65–74 years. Patients tend to be asymptomatic until rupture, which is often fatal.^1^

National ultrasound screening programmes for high-risk patients have significantly reduced deaths from rupture by identifying asymptomatic subjects with large aneurysms for elective surgical or endovascular repair. Once under surveillance, aneurysm diameter is monitored by serial ultrasound, the frequency of which is determined by baseline aneurysm size.^2^ Repair is recommended when the diameter exceeds 5.5 cm or where expansion is rapid. Importantly, smaller aneurysms (3.0–5.5 cm) still account for a fifth of all ruptures, and some aneurysms can greatly exceed 5.5 cm without rupture, suggesting aneurysm size is not the only determinant of rupture.^2 3^ To improve risk stratification in patients with AAA, a better understanding of the pathobiology of the disease is needed.^2^

Imaging techniques can measure arterial inflammation and calcification in atherosclerosis and have potential for use in aneurysm disease.^4–7^ 18-Fluorine-labelled 2-deoxy-2-fluoro-D-glucose (18F-FDG) positron emission tomography–CT (PET-CT) is commonly used for risk stratification in cancer. This technique has been adapted to measure vascular metabolic activity and provides a reproducible, non-invasive measure of arterial inflammation, reflecting glucose uptake by macrophages and other plaque cells.^8 9^

Histologically, aneurysms are associated with inflammatory infiltration, smooth muscle cell apoptosis and matrix degradation.^10^ These changes lead to weakening of the aortic wall, allowing expansion and rupture to occur. Although aneurysms are most common in the abdominal aorta, it has been suggested that the entire arterial system is abnormal in susceptible subjects, with dilatation of the carotid arteries being frequent in these patients.^11^Indeed, the incidence of popliteal aneurysms in patients with AAA is in the range of 10%–20%.^12^

Atherosclerosis and aneurysm disease frequently co-exist and share some underlying risk factors and pathologies, including calcification and inflammation, but the magnitude and regional distribution of these is not known. In addition, it is not clear what role thrombus plays, when present, within an aneurysm. Conflicting reports suggest both protective and deleterious effects.^13 14^

It has been suggested that both inflammation and calcification of the aneurysm wall are risk factors for aneurysm expansion and destabilisation.^4 15^ Although some retrospective series have been published, what have been lacking are simultaneous, non-invasive measurements of inflammation and calcification in aneurysm subjects, using state-of-the-art imaging protocols, and comparison with matched atherosclerotic controls without aneurysms.

To investigate these unanswered questions, this study aimed to determine the extent of inflammation and calcification in AAA and atherosclerosis using non-invasive imaging markers. We hypothesised that both aortic inflammation and calcification would be greater in aneurysm subjects than in matched controls with atherosclerosis, given that atherosclerosis is a disease largely confined to the intima, whereas AAA is a transmural disease. We also hypothesised that inflammation in aneurysms would not be restricted to the dilated region of aorta, but would instead demonstrate a global pattern throughout the entire aorta, and that thrombus would be associated with low levels of aneurysm inflammation.

## Methods

### Subjects

Patients were recruited from Addenbrooke’s Hospital, Cambridge and the Royal Infirmary of Edinburgh into two cohorts: (1) patients with asymptomatic AAA, and (2) age-and-sex-matched control patients with atherosclerosis but no aortic aneurysm disease (aneurysm was excluded using the non-contrast CT-derived aortic diameter). All control subjects were recruited in Cambridge, with patients with aneurysm recruited at both sites. The inclusion criteria for the study cohort were age >50 years and presence of an aneurysm between 3.0 and 5.5 cm on ultrasound. The inclusion criteria for the control cohort were clinically stable (>6 months) cardiovascular disease (defined as previous myocardial infarction, stroke or peripheral vascular disease). Exclusion criteria for both cohorts were type 1 or type 2 diabetes with a fasting glucose of >11 mmol/L, renal impairment (serum creatinine >250 µmol/L), contrast allergy or inability to provide informed consent.

### PET and CT imaging

All patients underwent 18F-FDG PET-CT imaging of the entire aorta. In addition, patients in the aneurysm group underwent contrast-enhanced CT imaging of the aorta. We used validated, reproducible imaging protocols.^7 16–18^

#### Patient preparation

Patients fasted for 6 hours prior to PET imaging.

#### Image acquisition and reconstruction

In Cambridge, a GE Discovery 690 PET-CT scanner was used. In Edinburgh, an equivalent Siemens machine was used (Biograph mCT, Siemens Medical Systems, Erlangen, Germany). A target dose of 240 MBq 18F-FDG was injected intravenously, after which patients rested in a quiet environment for 90 min before being transferred to the scanner. A non-contrast-enhanced attenuation correction CT scan (40 mAs per rotation (CareDose), 100 kV) was then performed followed by PET scan covering three bed positions from the arch of aorta to the aortic bifurcation over 30 min (10 min per bed position). Tracer circulation times were based on previous studies using 18F-FDG in atherosclerosis^7 16–18^ and aimed to produce optimal contrast between the aortic wall and the blood pool. In aneurysm subjects, with the patient in the same position, a CT aortogram from the diaphragm to the aortic bifurcation was performed using 75–100 mL of iodinated contrast (400 mgI/mL; Iomeron, Bracco, Milan, Italy), followed by 50 mL of 0.9% saline flush. The PET data were reconstructed using standard time of flight reconstruction algorithms. Corrections were applied for attenuation, dead time, scatter and random coincidences.

### Image analysis

#### PET-CT

Anonymised PET-CT datasets were analysed using an OsiriX workstation (64 bit; OsiriX Imaging Software, Geneva, Switzerland).^7 16–18^ PET images were fused with corresponding CT datasets, and regions of interest (ROIs) drawn on serial axial slices. Within these regions, mean and maximum tracer activities were measured using standard uptake values (SUV; the decay corrected tissue concentration of the tracer divided by the injected dose per body weight) and corrected for blood pool activity in the superior vena cava to provide tissue-to-background ratios (TBRs).^7 16–18^ The average of the maximum TBR values within each slice was expressed as the mean TBR_max_.

The aorta was divided into five segments for comparison: ascending aorta, descending thoracic aorta, abdominal non-aneurysmal aorta, aneurysm shoulder and aneurysm sac. The ascending aorta was defined as the segment of the aorta from the lower level of the right pulmonary artery up to the last slice where the aorta maintained its circular cross-sectional appearance. The descending thoracic aorta was defined as the region extending down from the circular slice below the arch of aorta to the slice where the diaphragm was first visible. The non-aneurysmal abdominal aorta was defined as the region between the descending aorta and the start of the aneurysm. The aortic aneurysm was defined as the region of abdominal aorta with all slices having a diameter of at least 3 cm. The aneurysm shoulder was defined as that segment of aorta bordering the first slice of aneurysmal aorta (two slices above and below the first 3 cm diameter slice). The aneurysm sac comprised the region between the aneurysm shoulder and the inferior aspect of the aneurysm.

Thrombus within the aneurysm was identified on the contrast CT aortogram using previously published Hounsfield unit definitions^19^ and sequential ROIs were drawn, avoiding overspill both from the lumen and aneurysm wall.

#### CT calcium scoring

Calcium scoring was performed using a CT workstation (Vital Images, Minnetonka, Minnesota, USA). Total Agatston scores were recorded for the entire aorta, using a threshold of 130 Hounsfield units for calcium on the non-contrast attenuation CT scan.^20^ In addition, arterial calcium scores, corrected for length of artery evaluated (in Agatston units per centimetre), were generated to allow comparison of aneurysmal segments in the aneurysm group and equivalent length segments of non-aneurysmal aortae in control subjects.

### Reproducibility studies

PET-CT data from 10 patients were selected at random to test the reproducibility of the 18F-FDG PET analyses. Two readers analysed the scans independently to provide a measure of the interobserver agreement for 18F-FDG uptake.

### Statistical analysis

Statistical analysis was performed using GraphPad Prism V.6 (GraphPad Software, USA) or SPSS V.19.0 where appropriate. Continuous data were checked for normality using the D’Agostino-Pearson omnibus test. Parametric variables are expressed as means±SD and compared using Student’s t-tests or repeat measure one-way analysis of variance with Tukey’s multiple comparison test when appropriate. Non-parametric data are presented as medians and IQRs and compared with the Mann-Whitney test, Wilcoxon matched-pairs signed rank or Friedman test as appropriate. Interobserver reproducibility was estimated using the Bland-Altman method and presented as mean bias ±2 SD, and intraclass correlation coefficients (ICC) with 95% CI. A two-sided p<0.05 was taken as statistically significant.

## Results

Sixty-three patients with AAA and 19 age-and-sex-matched subjects with atherosclerosis and no aneurysm were recruited. In one patient, PET-CT acquisition was not completed according to protocol—that subject was therefore excluded from analysis as the imaging data were incomplete.

The aneurysm and atherosclerosis groups were well-matched for age, sex and other cardiovascular risk factors (table 1). The mean aneurysm diameter was 4.33±0.73 cm.

**Table 1:** Baseline characteristics of all aneurysm and control subjects

Aneurysm group (n=63)	Controls (n=19)
*Demographics*		
Male/female	56/7	17/2
Age, years	76.1±6.8	69.4±5.8
Aneurysm diameter, cm	4.33±0.73	–
*Risk factors*		
Current or ex-smoker	47 (75%)	14 (74%)
Hypertension	26 (42%)	7 (37%)
Type two diabetes mellitus	5 (8%)	1 (5%)
Prior MI	14 (22%)	11 (58%)
Prior stroke	8 (13%)	4 (21%)
Prior PVD	9 (14%)	4 (21%)
*Medications*		
Aspirin	48 (76%)	16 (84%)
Statin	53 (84%)	19 (100%)
Beta-blocker	21 (33%)	10 (53%)
ACEI or ARB	30 (48%)	13 (68%)

ACEI, ACE inhibitor; ARB, angiotensin-receptor blocker; MI, myocardial infarction; PVD, peripheral vascular disease.

### Inflammation results

#### Inflammation in aneurysm and atherosclerosis groups

Compared with age-and-sex-matched patients with atherosclerosis, patients with aneurysms had higher 18F-FDG uptake across their entire aorta (mean TBR_max_ 2.16±0.51 vs 1.70±0.22, p=0.001). Greater inflammation was also noted in all individual aortic regions of aneurysm subjects compared with atherosclerotic controls (table 2).

**Table 2:** Comparison of inflammation and calcification in the aneurysm and matched control groups

	Aneurysm subgroup (n=19)	Matched controls (n=19)	P value
Ascending aorta			
Mean TBR_max_	2.14±0.53	1.84±0.27	0.038
Agatston score (median (IQR))	18 (0–295)	0 (0–42)	0.246
Agatston score/cm	6 (0–62)	0 (0–9)	0.218
Descending aorta			
Mean TBR_max_	2.15±0.58	1.70±0.24	0.005
Agatston score (median (IQR))	267 (0–1230)	320 (170–842)	0.435
Agatston score/cm	25 (0–72)	23 (16–67)	0.617
Abdominal aorta (incl. aneurysm)			
Mean TBR_max_	2.23±0.45	1.68±0.21	<0.0001
Agatston score (median (IQR))	4483 (3105–9430)	2879 (941–7270)	0.112
Agatston score/cm of abdominal aorta	245 (148–408)	166 (80–374)	0.307
Agatston score/cm in aneurysm and control equivalent	442 (304–920)	166 (80–374)	0.004
Entire aorta			
Mean TBR_max_	2.16±0.51	1.70±0.22	0.001
Agatston score (median (IQR))	5136 (3297–9360)	3735 (1425–8261)	0.180
Agatston score/cm	144 (87–266)	109 (49–219)	0.230

#### Regionality of inflammation within aneurysm subjects

**Figure 1:**
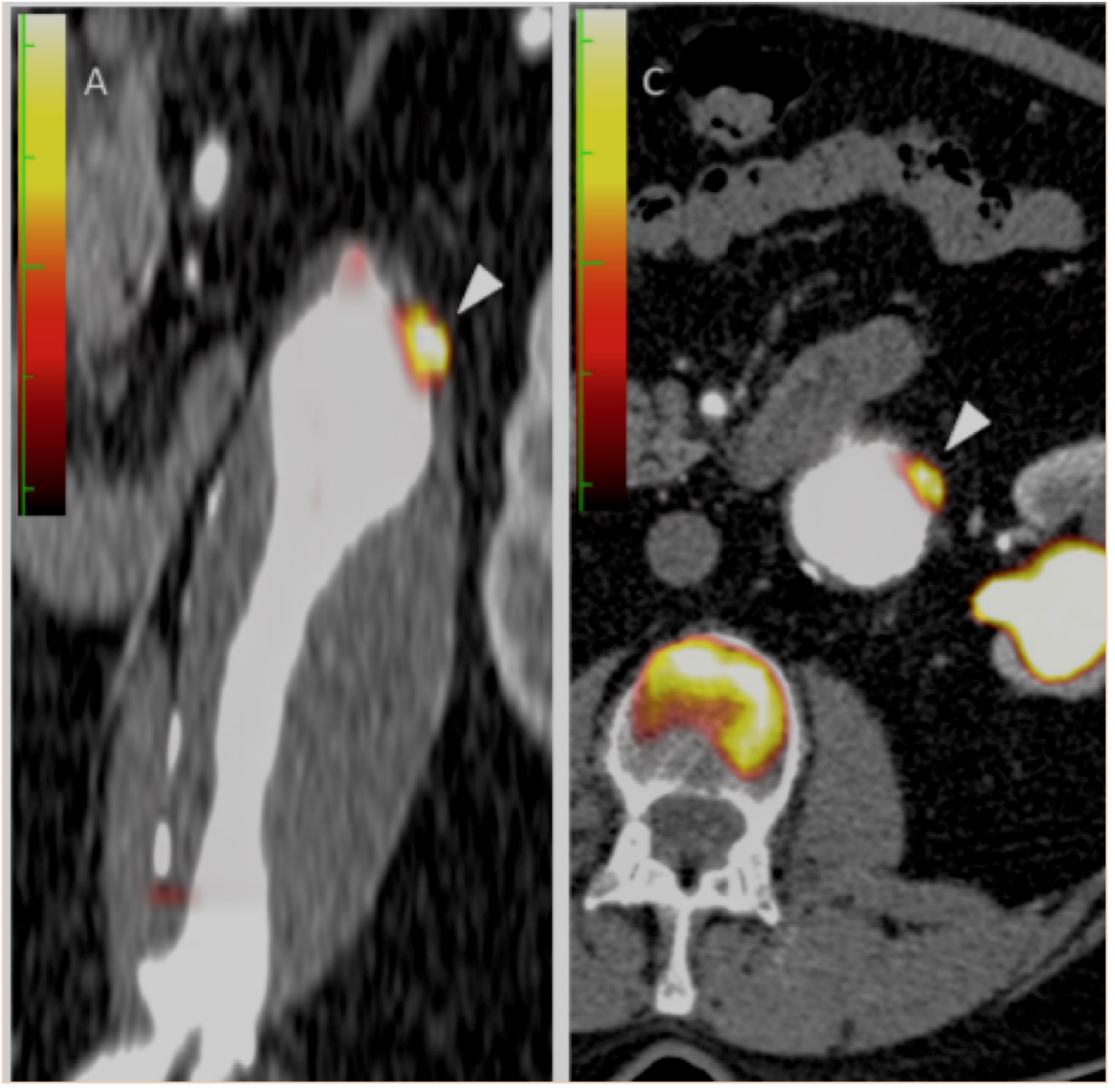
Coronal and transaxial fused positron emission tomography and contrast CT images demonstrating focal 18F-FDG uptake within the aneurysmal wall (white arrowheads). Note also the calcified lateral aspect of the aneurysm.

**Figure 2:**
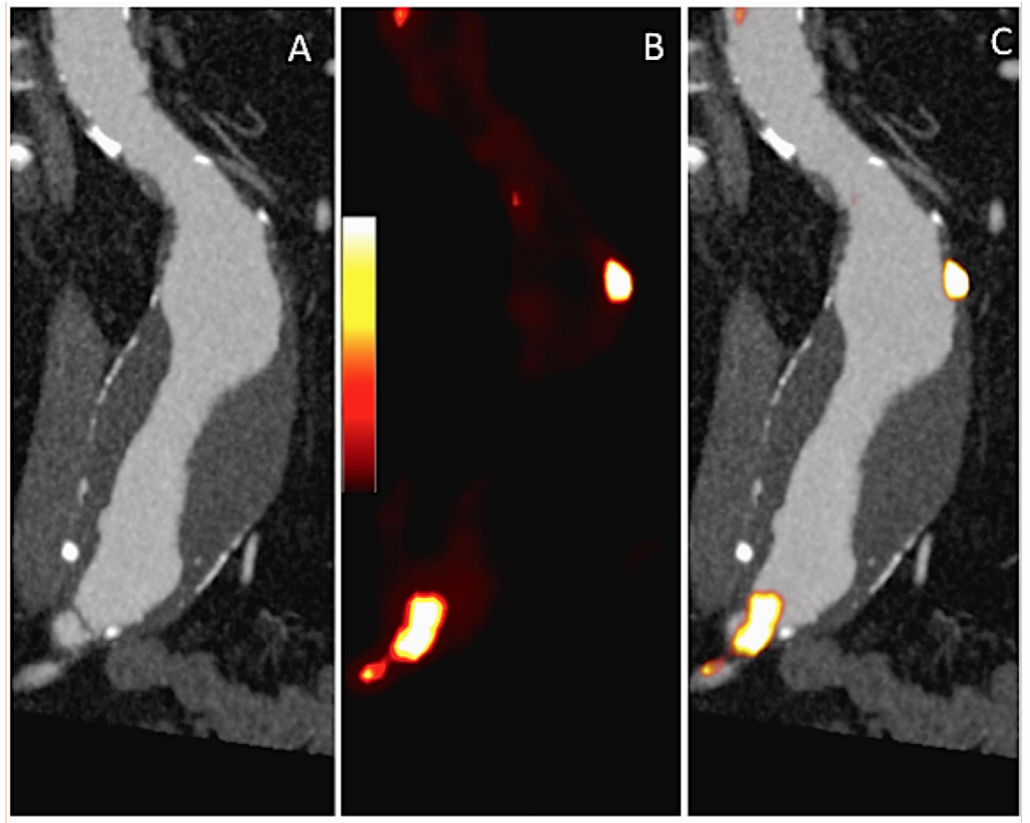
Coronal contrast CT, 18F-FDG positron emission tomography (PET) and fused images from a subject with a 4.5 cm aneurysm demonstrating focal 18F-FDG uptake within the aneurysmal wall (golden colour on the PET images). Note also the presence of intraluminal thrombus and calcification.

Among the aneurysm group, 18F-FDG accumulation was greater within the aneurysm itself than in the non-aneurysmal segments of the aorta (mean TBR_max_ 2.23±0.46 vs 2.12±0.46, p=0.024, figures 1 and 2 and table 3). Exploring further, 18F-FDG uptake was consistently higher in both the sac and the shoulder regions of the aneurysm than in any non-aneurysmal segment of the aorta (p=0.0004, table 4).

**Table 3:** Inflammation and calcification in aortic aneurysm (n=63), comparing aneurysmal and non-aneurysmal aortic regions

	Aneurysmal aorta (n=63)	Non-aneurysmal aorta (n=63)	P value
Mean TBR_max_	2.23±0.46	2.12±0.46	0.024
Agatston score (median(IQR))	4918 (2901–8008)	1017 (139–2226)	<0.0001

**Table 4:** Inflammation and calcification in aortic aneurysm (n=63), with values for each individual region of the aorta

	Shoulder	Sac	Ascending	Descending	Abdominal	P value
Mean TBR_max_	2.26±0.49	2.25±0.46	2.12±0.41	2.15±0.38	2.13±0.38	0.0004
Agatston score (median(IQR))	342 (109–7441)	4436 (2554–7441)	50 (0–674)	549 (118–1606)	1575 (717–3275)	<0.0001

Inflammation (mean TBR_max_) and calcification (Agatston score) distribution in aneurysm subjects.

#### Association between inflammation and AAA size

There was no overall correlation between aneurysm size and 18F-FDG uptake within its wall (r=0.12, p=0.36). However, when divided into two groups by average aneurysm diameter (4.33 cm), there was a non-significant trend toward more 18F-FDG uptake in smaller compared with larger aneurysms (mean TBR_max_ 2.36±0.45 vs 2.14±0.45, n=32 vs n=30, p=0.065).

#### The effect of aneurysm thrombus

Intraluminal thrombus was present in 43 of patients with aneurysm (69%). Subjects with no thrombus had higher 18F-FDG uptake within their aneurysmal walls when compared with those with thrombus (mean TBR_max_ aneurysm 2.43±0.45 vs 2.14±0.43, p=0.018). 18F-FDG uptake within the thrombus itself was consistently lower than in any region of the aortic wall in these subjects (mean TBR_max_ within thrombus 1.30±0.48 vs aneurysm wall 2.23±0.46, p<0.0001).

### Reproducibility of 18F-FDG measurements

The interobserver reproducibility of 18F-FDG measurements was excellent across all aortic regions, including the aneurysm. Across the aorta as a whole, the Bland-Altman limits of agreement for mean TBR_max_ were 0.04±0.07. Intraclass correlation coefficients were >0.90, with the shoulder and sac of the aneurysm having the lowest ICC values at 0.91 and 0.96, respectively.

### Calcification results

#### Calcification in aneurysm and control subjects

In the aneurysm group, calcification was greater within the aneurysmal aortic wall than in the non-aneurysmal segments of aorta (4918 (2901–8008) vs 1017 (139–2226) Agatston units, p<0.0001). The extent of calcification was higher in the aneurysm sac than in all other aortic regions (p<0.0001, table 4). Consistent with these findings, aortic aneurysms were more calcified than the corresponding regions of abdominal aorta in atherosclerotic controls (442 Agatston units per cm (304–920) vs 166 (80–374), p=0.0042, table 2). Of note, no differences in calcification were noted between aneurysm subjects and controls in any other aortic region. No significant relationships were observed between aneurysm diameter and extent of calcification (r=0.08, p=0.53). No differences were observed in the extent of wall calcification in those with or without intraluminal aneurysm thrombus (5310 (2748–9165) vs 4903 (2952–7856), p=0.57).

### Relationship between inflammation and calcification in AAA and atherosclerosis

There was no correlation between inflammation and calcification within aneurysms (r=−0.153, p=0.235). Similarly, in the control group with atherosclerosis, there was no correlation between aortic inflammation and calcification (total aorta: r=−0.19, p=0.45; abdominal aorta: r=−0.13, p=0.6).

## Discussion

PET imaging to measure inflammation within aortic aneurysm was first reported in 2002.^6^ Our study is the first to measure both inflammation and calcification in patients with aneurysm and to compare the results with well-matched controls with atherosclerosis.

We found that patients with asymptomatic aortic aneurysms had excessive inflammation within the aneurysmal segment of the aorta. Intriguingly, compared with matched controls with atherosclerosis, the entire aorta in those with an aneurysm was more highly inflamed. This suggests the presence of a global inflammatory aortopathy rather than a disease simply confined to the abdominal aorta. We also found that intraluminal thrombus, a frequent feature of aneurysms, was metabolically inert, with little 18F-FDG uptake in most of our cases.

Calcification was also greatest in the aneurysmal part of the aorta in the aneurysm group and far more prevalent in patients with aneurysm than matched atherosclerotic subjects. Interestingly, in contrast to the global increase in aortic inflammation demonstrated in patients with aneurysm, calcification was not exhibited more than in controls in non-aneurysmal regions of their aortae. Further, there was also no correlation between the degree of calcification and inflammation in either cohort. This suggests that, while inflammation and calcification are two important biological processes implicated in aneurysm disease, they are distinct elements. Perhaps the global inflammation seen throughout the aorta represents an abnormal vasculature prone to aneurysmal dilatation, whereas calcification is more specific to the aneurysmal and biologically active portion of the vessel. Certainly, there is a suggestion that inflammation and calcification occur at different stages of the atherosclerotic disease process.^21^

While there was no significant relationship observed between baseline aneurysm size and 18F-FDG uptake, consistent with a previous report^22^ smaller aneurysms appeared more 18F-FDG-avid than larger ones. This might be explained by a scenario where intense, early inflammation causes mechanical weakening of the aortic wall, allowing expansion to occur. Inflammation then reduces as the wall becomes more calcified. Indeed, there is good evidence that inflammation plays a crucial role in AAA development, and some have postulated that the anti-inflammatory actions of statins may be beneficial in reducing AAA growth.

### What is the relationship between aortic aneurysm and atherosclerosis?

Aortic aneurysms frequently occur in patients with atherosclerosis. The two disease processes share several common risk factors, notably cigarette smoking.^23^ The underlying pathologies of the two conditions overlap to an extent, with inflammation and calcification being common to both. Nevertheless, there are important differences. Diabetes seems to protect against aneurysm formation and growth.^23^ While aneurysms are characterised by weakening of the media of the aorta by chronic inflammation and degradation of the extracellular matrix,^24^ in atherosclerosis the main insult, at least initially, is to the intima of the artery. In atherosclerosis, a fibrous cap is typical over the necrotic core of the lesion, whereas in aneurysm, there is very often a large adherent thrombus, variously described as protective against expansion in some studies^25^ and encouraging destabilisation in others.^26 27^ The role of calcification is also debated in both conditions.^15^ In atherosclerosis, macrocalcification is thought to be a healing response conferring plaque stability, but microcalcification appears to be associated with a risk of plaque rupture.^28^

Although calcification within aneurysms was a common feature, its role is less well understood than in atherosclerosis. It is unclear whether aneurysm-associated macrocalcification is a high-risk feature, suggestive of active, intense, necrotic inflammation, or whether it represents a burned-out pacified process following previous inflammation. Data from the prospective SoFIA3 study highlighted the ability of 18F-fluoride PET to detect active aneurysmal microcalcification, with aneurysms with higher 18F-fluoride uptake having 2.5 times more rapid aneurysm expansion, and being nearly three times more likely to experience AAA repair or rupture, compared with patients with less aneurysmal microcalcification.^29^ Whether the same holds true for macrocalcification remains unknown. Furthermore, the processes that govern calcification within the aneurysmal and the non-aneurysmal segments of aorta within aneurysm patients may well be distinct.

In terms of whether 18F-FDG uptake predicts future aneurysm behaviour, published work presents a mixed picture. There is evidence to suggest that 18F-FDG can discriminate between asymptomatic and symptomatic aneurysms, but its potential as a marker of aneurysm expansion and rupture has yet to be established.^22 30–32^ There do appear to be positive correlations between the extent of 18F-FDG uptake and the degree of mechanical wall stress within an aneurysm.^33^ Considerably more work is needed to determine whether data derived from advanced imaging, such as the extent of inflammation and calcification, can improve clinical decision-making and our understanding of this common condition.

## Conclusion

Our study adds several important mechanistic insights into the pathobiology of AAAs. We observed that both aortic inflammation and calcification are greater in patients with aortic aneurysm than those with atherosclerosis alone, that aortic inflammation typically extends beyond the aneurysmal sac to involve the entirety of the aorta and that greater thrombus burden is associated with less aneurysm inflammation. Future studies are needed to evaluate the prognostic value of measuring aortic inflammation and calcification to improve clinical decision-making in patients with asymptomatic AAA.
