# Correction: Greater aortic inflammation and calcification in abdominal aortic aneurysmal disease than atherosclerosis: a prospective matched cohort study

**DOI:** 10.1136/openhrt-2019-001141corr1

**Published:** 2020-07-30

Joshi NV, Elkhawad M, Forsythe RO, *et al*. Greater aortic inflammation and calcification in abdominal aortic aneurysmal disease than atherosclerosis: a prospective matched cohort study. *Open Heart* 2020;7:e001141. doi: 10.1136/openhrt-2019-001141.

Author name Jonathan Boyle has been updated to Jonathan R Boyle.

